# Skip the Trip: Air Travelers' Behavioral Responses to Pandemic Influenza

**DOI:** 10.1371/journal.pone.0058249

**Published:** 2013-03-20

**Authors:** Eli P. Fenichel, Nicolai V. Kuminoff, Gerardo Chowell

**Affiliations:** 1 Yale School of Forestry and Environmental Studies, New Haven, Connecticut, United States of America; 2 Department of Economics, Arizona State University, Tempe, Arizona, United States of America; 3 School of Human Evolution and School Change, Arizona State University, Tempe, Arizona, United States of America; 4 Division of Epidemiology and Population Studies, Fogarty International Center, National Institutes of Health, Bethesda, Maryland, United States of America; University of Oxford, Vietnam

## Abstract

Theory suggests that human behavior has implications for disease spread. We examine the hypothesis that individuals engage in voluntary defensive behavior during an epidemic. We estimate the number of passengers missing previously purchased flights as a function of concern for swine flu or A/H1N1 influenza using 1.7 million detailed flight records, Google Trends, and the World Health Organization's FluNet data. We estimate that concern over “swine flu,” as measured by Google Trends, accounted for 0.34% of missed flights during the epidemic. The Google Trends data correlates strongly with media attention, but poorly (at times negatively) with reported cases in FluNet. Passengers show no response to reported cases. Passengers skipping their purchased trips forwent at least $50 M in travel related benefits. Responding to actual cases would have cut this estimate in half. Thus, people appear to respond to an epidemic by voluntarily engaging in self-protection behavior, but this behavior may not be responsive to objective measures of risk. Clearer risk communication could substantially reduce epidemic costs. People undertaking costly risk reduction behavior, for example, forgoing nonrefundable flights, suggests they may also make less costly behavior adjustments to avoid infection. Accounting for defensive behaviors may be important for forecasting epidemics, but linking behavior with epidemics likely requires consideration of risk communication.

## Introduction

There has been a rapid rise in interest in how behavioral change in response to the risk of contracting an infectious disease influences epidemiological dynamics and public health outcomes [1]–[5]. Measuring behavioral responses is important for testing theories and for informing public health policy. Moreover, measuring behavioral responses is important in its own right to evaluate public health communication strategies and measure the cost of epidemics. One type of response that may be particularly important is a change in the behavior of air travelers [6].

Air travelers have the potential to rapidly spread infectious disease over long distances [7], [8]. Moreover, air travel is perceived to be a risky behavior with respect to infectious diseases such as influenzas. There has been considerable interest in the role of air travel, and possible air travel restrictions, on the spread of an infectious disease such as avian or 2009 A/H1N1 influenza, commonly called swine flu [7], [9], [10]. Previous studies have concluded that the net benefits to society of air travel restrictions are at best small [11]–[13], but most models rely on assumptions that have not been confronted with data [14]. What does seem clear is that flying increases the likelihood of air travelers contracting an infectious disease conditional on there being on infectious person on the airplane [15], [16], and that the public links flying with infection risk. Air travel may increase private infection risk beyond an acceptable level, and public health interventions related to travel and airport surveillance programs may benefit travelers [17], [18].

In this paper, we take advantage of the high cost of flying and the high perceived risk of infection associated with flying along with a novel dataset of flight records to investigate whether travelers changed their travel decisions in response to a pandemic influenza. Then we quantify a lower bound on the cost of such adjustments. We also contrast travelers' responsiveness to subjective measures of public concern for the epidemic with their responsiveness to objective measures of the state of the epidemic.

Air travel is a unique activity. Tickets are often purchased weeks in advance, are non-refundable and non-transferable, and represent non-trivial expenditures. Economic theory suggests that an individual values a trip at a value at least as great as the price paid for the ticket. It follows that individuals who choose not to use a pre-paid ticket, as a result of an ongoing infectious disease epidemic, value the perceived reduction in infection risk at least as much as the cost of canceling their flight. Travelers who use their tickets may still value a reduction in health risk, but the value they assign to this reduction must be less than the cost of forgoing a pre-paid trip. Johansson et al. [14] assert that the value of a trip is often much greater than the health benefits of forgoing a trip and assume that infection risk does not affect flying behavior. In this paper, we test the hypothesis that indices of infection risk influenced the number of passengers changing travel plans, forgoing pre-paid tickets, and missing flights using data from 1.7 million flights operated by a major US air carrier.

For people to adapt their behavior to an epidemic, they must be informed about the epidemic. Prior research focused on how the media and information influence epidemics [19]–[22]. We use a Google Trends index of the intensity of internet searches for the phrase “swine flu” as a potential proxy for the public's perceived risk of infection. Google Trends is distinct from Google Flu Trends. Google Trends has been shown to track public sediment in other social areas, such as consumer confidence [23]. Furthermore, the search index we use is highly correlated with the volume of news stories on the swine flu epidemic (*R = *0.98), as reported by Google. We compare travelers' responses to the Google Trends index to travelers' responses to the number of reported confirmed A/H1N1 cases in the World Health Organization's FluNet database.

Our results suggest that a subset of passengers, who had already purchased tickets, chose not to fly in response to swine flu. Equally important, we demonstrate that this avoidance behavior did not track the incidence curve of reported confirmed A/H1N1 cases. Travelers appear to have responded to the incidence of media attention to swine flu, which was generally weakly, and at times negatively, correlated with objective measures of risk. We use data on flight prices to estimate a lower bound of the value of eliminating infection risk to air passengers, and a lower bound on the value of communications strategies that more accurately track cases. Our analysis has two broad implications. First, some people make costly changes in behavior to avoid infection, even without government mandated policies for social distancing [24]–[28]. It follows that people may also make less costly adjustments without government mandates, and these behavioral adjustments may influence disease dynamics. Such behavioral changes would make current approaches to estimating transmission parameters biased and inconsistent [29]. Second, media attention to epidemics is a poor measure of actual cases, and can lead to mal-adaptive behavior. This finding suggests that there is value in developing easily accessible sources of spatially delineated information on the localized risk of contracting infectious diseases (e.g. websites or apps reporting infection rates by city).

## Methods

### The data

U.S. Airways Corporation provided access to proprietary data on individual coach (economy) class flights covering all flights from April 1, 2008 to March 31, 2010. Equivalent data are available from IATA, www.iata.org. U.S. Airways was the fifth largest commercial carrier in the United States during this period and had a 7.22% market share (U.S. Department of Transportation, Research and Innovation Technology Administration, http://www.transtats.bts.gov). U.S. Airways offers extensive connections within the United States as well as international connections to Mexico, the Caribbean, Latin and South America, Europe, Israel, and Canada. The U.S. Airways customer base consists almost entirely of U.S. residents. For each flight, we observe the number of passengers booked as of 24 hours prior to departure, the number of passengers flown, the number of passengers with connecting flights, aircraft seating capacity, the mean and median prices paid by coach passengers on each flight, the flight's origin, and its destination. Table 1 presents summary statistics for the dataset used in our analysis.

**Table 1:** Summary statistics main variables of interest.

Variable	Observations	Mean	Standard Deviation	Min	Max
booked passengers per flight	1,659,974	71	44.97	1	296
passengers flown per flight	1,659,974	66	42.51	1	262
mean price ($)	1,659,974	172	63.63	7	5722
median price ($)	1,659,974	150	63.86	4	1190
passengers connecting from other flights	1,659,974	25.76	33.89	0	258
number of passengers missing flights	1,659,974	4.64	5.39	0	107
Google Trends swine flu index	106	8.67	30.19	0	294
Google Trends swine flu index 2 week moving average	730	8.65	22.86	0	183
FluNet cases	106	1,030	1,885.26	0	9,735
FluNet cases 2 week moving average	730	1,028	1,852.10	0	9,181

**Figure 1:**
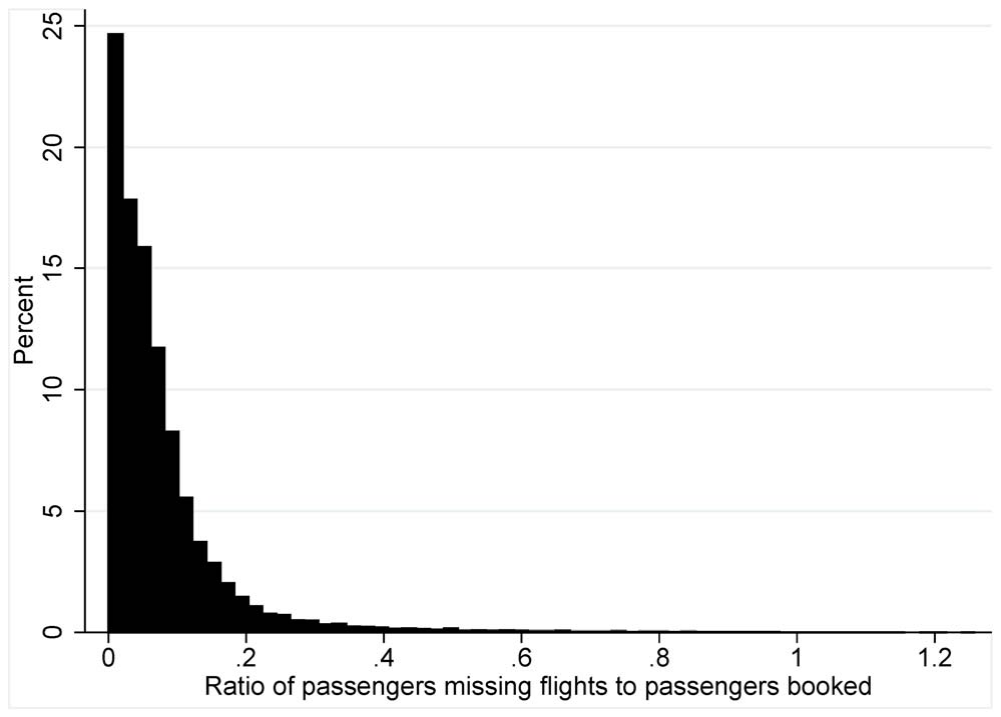
Distribution of the proportion of booked passengers missing flights for flights with excess capacity.

The original data provided by U.S. Airways came from gate agent records, and these records are not always entered correctly when aircrafts change and passengers are rebooked by the airline at the last minute. As a result there were some obvious coding errors. Such records were removed prior to beginning analysis (removed records are not included in the statistics in Table 1); e.g., negative passengers flown or zero capacity, a median ticket price per flight of zero, or where more than 40% of the booked passengers missed the flight. A large share of passengers missing a flight could occur when a large number of passengers rebook on a flight to the same destination when the originally booked flight was substantially delayed. The 40% threshold was chosen by visually inspecting the distribution of the proportion of passengers missing flights and substantial support dissipates around 40% (Figure 1). 40% was 3.6 standard deviations above the mean. Overall our measure of missed flights corresponds to a lower bound on the number of passengers missing flights. We focused our research on flights with at least one available seat to avoid data truncation issues.

U.S. Airways flies 20 different aircraft types that can be classified into groups of aircraft that are used for qualitatively similar types of flights. Aircraft types can be recovered from the aircraft seating capacity reported in each flight record (http://www.usairways.com/en-US/aboutus/pressroom/fleet.html). We grouped all aircrafts into seven categories: small express (≤50 seats); large express (50–87 seats); small mainline (88 seats); standard mainline (89–138 seats); large mainline (139–176 seats); small international (177–188 seats); and large international (>188 seats). We classified flight destinations into eight regions: the continental US, Hawaii, Alaska, Mexico, Canada, Latin and South America, the Caribbean, and Europe and Israel.

**Figure 2:**
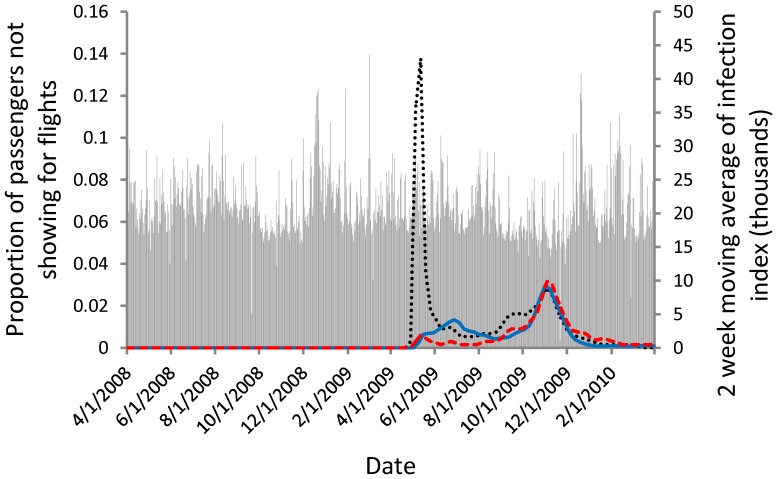
The proportion of passengers missing flights (grey bars), the two-week moving average of FluNet reported cases (blue solid line), and the two-week moving average of Google Trends swine flu index (black dotted line) and H1N1 index (red dashed line). Google Trends indices are scaled by 235 for easy comparison with the FluNet data. A graph with the total of passengers missing flights as opposed to proportion of passengers looks qualitatively similar. Online version in color.

We investigated two measure of the A/H1N1 epidemic. First, the Google Trends index of browser search intensity for the phrase “swine flu” (Fig 2). In prior work, Google Flu Trends, an index created by Google to track flu, has been shown to follow closely influenza epidemics in the United States [30], [31] and, after an adjustment, the A/H1N1 epidemic [32]. Our purpose is slightly different. We wish to measure public knowledge about the epidemic, and an index of searches for information should be a good proxy measure of concern. It is important to note that concern does not have to correlate with actual cases. “Swine flu” was the most searched news story on Google in the spring quarter of 2009 (http://www.google.com/intl/en_us/press/zeitgeist2009/overview.html), and “swine flu” was the third top trending search on Microsoft Bing in 2009, behind only “Michael Jackson” and “Twitter”. Our second measure is reported A/H1N1 cases in the World Health Organization's FluNet database, http://gamapserver.who.int/GlobalAtlas/home.asp (Fig 2).

Google and FluNet data are only available at the weekly time step so the data had to be smoothed to apply measurements to flights on specific days. Also, we are unsure of the time frame during which passengers would have chosen to abandon their original flight plans. We consider different approaches to matching the weekly Google Trends and FluNet data to the daily flight data; only applying the index value for the given day applied from its week, a two-week (Fig 2), and three-week moving average.

### Estimating the effect of the epidemic

The number of passengers missing a flight was measured as the difference between the number of passengers booked on a flight and the number of passengers who actually flew. This measure of missed flights is derived from passenger counts and ranges from 0 to 107 in our data. Missed flights can be affected by many factors aside from concern for infection. Therefore, we statistically controlled for several potential confounders, including day of the week, month, ticket price, numbers of passengers booked and connecting passengers, aircraft class, and airport weather data. We also included airport specific fixed effects to help control for unobserved features of weather that may be correlated with the season or geography.

A natural approach to modeling the count of passengers missing a flight is to assume a Poisson distribution and use a generalized linear model; however data are often over-dispersed in practice. The negative binomial regression generalizes the Poisson regression to allow for over-dispersion [33], [34]. Despite this generalization, heteroskedasticity in the error terms can still persist, and we expect that errors may be correlated within origination airport. This was addressed by using cluster-robust standard errors that allow unknown forms of heteroskedasticity as well as unknown forms of correlation among the error terms associated with each origination airport ([33] pp. 569–579). Negative binomial models were fit by maximum (pseudo) likelihood using Stata 12. Pseudolikelihoods were used to accommodate the cluster robust standard errors because standard maximum likelihood underestimates the true variance with over-dispersion [35 p.683], when robust estimators are used for the variance-covariance matrix ([33] p. 574), and pseudolikelihoods are commonly required for generalized linear models ([35] p. 149–150). Using a negative binomial link function ([34] pp. 114–116, [35] p. 675) the linear component of the model consists of, β_0_+***W*** **β_1_**+***P*** **β_2_**+***R*** **β_3_**+***D*** **β_4_** +***M*** **β_5_**+***T*** **β_6_**+***L*** **β_7_**+***F*** **δ**+γ*I* where β_0_ is an intercept, **β** ***_i_*** *i* ∈{1,2…7} are vectors of parameters associated with a vector of airport weather fixed effects (dummy variables), ***W***; a vector of airplane type fixed effects, ***P***; a vector of destination regions fixed effects, ***R***; a vector of day fixed effects ***D***; a vector of month fixed effects ***M***; and vectors of fixed effects for the origination, ***T***, and destination airports ***L***. Flight specific variables are captured in vector ***F,*** and **δ** is an associated vector of coefficients. The subjective (objective) measure of the intensity of the epidemic, Google Trends (FluNet), is represented by *I*, and γ is a parameter measuring the effect of the epidemic.

To investigate the sensitivity of results to modeling assumptions, several versions of the model were estimated. For example, separate models were run in which price was defined as either the mean or median pre-paid ticket price for passengers sitting in coach. We also consider models with interaction terms between destination region and influenza index and interaction terms between destination city and influenza index. A total of 36 specifications of the model were considered. The variations included all permutations of the definition for the risk variable (Google Trends or FluNet data); the time step over which the risk variable is aggregated (a one-week, two-week or three-week moving average); the summary measure of ticket price on a given flight (mean or median); and the interactions included in the model (none, flu index by destination region, or flu index by destination city interaction terms).

### Calculating willingness to pay to avoid infection

U.S. Airways provided data to us at the aggregate flight level. We cannot track individual passengers. Based on the aggregate flight level data we compute a lower bound for the willingness to pay to avoid infection for the flying population. The lower bound for aggregate willingness to pay to avoid infection was computed by calculating the estimated number of missed flights attributed to concern about swine flu. This was done by taking the observed number of missed flights and subtracting the predicted number of missed flights in a counterfactual scenario where the A/H1N1 outbreak did not occur. To compute the counterfactual scenario, we forecasted flights missed with the influenza index set to zero for each flight in our data set. To arrive at a willingness to pay to avoid infection per flight, the number of passengers missing each flight in the data set, attributed to the epidemic, was then multiplied by the median price paid for that flight. The aggregate lower bound willingness to pay was then scaled up to represent the national airline market. The resulting measure is best interpreted as a lower bound for three reasons. First, passengers who bought tickets must have done so because they valued the flight at the purchase price or higher. Thus, abandoning the planned flight costs the passenger the purchase price, plus any surplus that the passenger would have received from taking the flight. Second, passengers who did not miss flights may have been willing to pay to reduce the probability of catching swine flu, but were only willing to pay less than the cost of skipping the flight. Finally, missing a flight is a relatively large consumer decision. If consumers make such extreme behavioral decisions, then it follows that they may also make additional, less costly, adjustments to daily routines influencing their social contacts which, in turn, may locally affect pathogen transmission dynamics.

## Results

### Google swine flu estimation results

The negative binomial count data model fit the data well for all specification at all standard confidence levels. For models that only included main effects, i.e., no interaction terms, the two-week moving average Google Trends index of internet search intensity for “swine flu” with median ticket prices had the greatest log pseudolikelhood (Table 2). This model is treated as the base specification, with results presented in Tables 3 and 4. Baseline results were found to be robust to most modeling decisions. Results for other specifications are discussed only when they differ from the base specification. Complete results from all specifications will be provided upon request.

**Table 2:** Model specification robustness results for Google Trend “swine flu” models.

Length of moving average	Price index	Regional interaction with flu index	City interactions with flu index	Log pseudo- likelihood	Estimate of flu index coefficient	p-value for coefficient
1	median	no	no	−4022253	−3.91×10^−6^	0.890
1	mean	no	no	−4022438	−1.37×10^−6^	0.964
2	mean	no	no	−4022386	4.14×10^−4^	0.067
2	median	no	no	−4022202	4.07×10^−4^	0.072
3	mean	no	no	−4022412	3.63×10^−4^	0.114
3	median	no	no	−4022228	3.53×10^−4^	0.131
1	median	yes	no	−4022188	−2.9 ×10^−5^	0.296
1	mean	yes	no	−4022373	−2.7×10^−5^	0.355
2	mean	yes	no	−4022303	3.97×10^−4^	0.069
2	median	yes	no	−4022119	3.92×10^−4^	0.073
3	mean	yes	no	−4022325	3.56×10^−4^	0.102
3	median	yes	no	−4022140	3.47×10^−4^	0.115
1	mean	no	yes	−4022125	7.6×10^−5^	0.289
1	median	no	yes	−4021937	7.43×10^−5^	0.33
2	mean	no	yes	−4021977	7.28×10^−4^	0.123
2	median	no	yes	−4021790	7.22×10^−4^	0.132
3	mean	no	yes	−4021967	7.46×10^−4^	0.229
3	median	no	yes	−4021780	7.38×10^−4^	0.238

When days are assigned their epidemic index value from weekly data, the length of moving average is listed as 1. Price index lists whether median or mean prices were used, the interactions columns state whether interactions were included. Coefficients for models that include interaction effects should be read with care, because some effect of the epidemic index is inputted through the interaction terms, which are not shown.

**Table 3:** Parameter estimates based on a negative binomial regression using median price and a two-week moving average of the Google Trends swine flu index (first part).

Variable	Estimate	Cluster robust Standard Error	Z-score	p-value
**Constant**	125.83950	21.28549	5.91	0
**Google Trends swine flu index 2 week moving avg**	0.00041	0.00023	1.8	0.072
**Flight specific variables**				
booked passengers	0.01406	0.00098	14.35	0
median price	−0.00066	0.00023	−2.91	0.004
inbound connections	−0.00360	0.00125	−2.89	0.004
**Weather variables**				
weather Phoenix (PHX)	−0.00791	0.01029	−0.77	0.442
weather Philadelphia (PHI)	0.08189	0.01891	4.33	0
weather Chicago (ORD)	0.01280	0.00209	6.12	0
weather Las Vegas (LAS)	0.00384	0.00691	0.56	0.578
weather New York (JFK)	0.08015	0.01601	5.01	0
weather Charlotte (CLT)	0.05706	0.01927	2.96	0.003
**Aircraft type**				
large express	0.02164	0.02416	0.9	0.371
small mainline	0.16461	0.02743	6	0
standard mainline	0.00489	0.03413	0.14	0.886
large mainline	−0.32912	0.05060	−6.5	0
small international	−0.48461	0.06032	−8.03	0
large international	−0.83046	0.06323	−13.13	0
**Destination region**				
Mexico	−0.11871	0.15826	−0.75	0.453
Latin & South America	−0.15440	0.16639	−0.93	0.353
Hawaii	−0.23949	0.16782	−1.43	0.154
Europe & Israel	0.21284	0.15120	1.41	0.159
Caribbean	−0.58451	0.22634	−2.58	0.01
Canada	−0.24884	0.06394	−3.89	0
Alaska	−2.14877	0.16360	−13.13	0

**Table 4:** Parameter estimates based on a negative binomial regression using median price and a two-week moving average of the Google Trends swine flu index (second part).

*Variable*	*Estimate*	*Cluster robust Standard Error*	*Z-score*	*p-value*
**Year**	−0.06266	0.01058	−5.92	0
**Day of the week**				
Monday	0.03281	0.00800	4.1	0
Tuesday	−0.01412	0.00485	−2.91	0.004
Wednesday	−0.04894	0.00491	−9.96	0
Thursday	−0.02388	0.00709	−3.37	0.001
Friday	0.02187	0.00881	2.48	0.013
Saturday	−0.06876	0.00842	−8.17	0
**Month**				
February	−0.02185	0.02021	−1.08	0.28
March	−0.05164	0.01547	−3.34	0.001
April	−0.07759	0.02262	−3.43	0.001
May	−0.12067	0.02927	−4.12	0
June	−0.06483	0.02259	−2.87	0.004
July	−0.07066	0.02115	−3.34	0.001
August	−0.02590	0.02292	−1.13	0.259
September	−0.17408	0.03113	−5.59	0
October	−0.21746	0.02348	−9.26	0
November	−0.25200	0.02300	−10.96	0
December	0.04681	0.01530	3.06	0.002
**Over-dispersion parameter**	0.53694	0.01942		

Correlations between observed and predicted values are the appropriate way to assess goodness of fit for count-data models [33]. The predicted missed flights showed a 0.32 correlation with observed missed flights, and a Spearman's rank correlation of 0.59. For both correlations the *p*-value<0.0001 (null hypothesis of no correlation), and correlations results were not meaningfully altered by alternative specifications. Tests for over-dispersion suggest that the over-dispersion parameter was significantly different from zero (Table 4).

The parameters for statistical controls were generally significantly different from zero and had the expected signs (Tables 3 and 4). For all of the specifications that we tested, passengers booked, and poor weather in airports CLT, PHI, JFK, and ORD had statistically significant positive effects on the number of missed flights. Weather in LAS and PHX did not have significant effects. This can be explained by the fact that poor weather is relatively rare in LAS and PHX, tends to occur later in the day, and has less scope to reverberate through the airline's network due to the 3-hour time difference with the east coast. Ticket price and number of inbound connecting passengers had significant negative effects, suggesting that passengers who paid more for flights were more likely to show up and that, all else equal, passengers in the system were less likely to miss a connecting flight. Passengers were significantly less likely to miss flights on aircrafts serving international routes or large domestic aircraft used on longer routes. Furthermore, passengers were more likely to miss flights on small aircrafts serving mainline flights. These commuter flights are dominated by business travelers. There are typically multiple connections a day for these routes; it is reasonable to expect that this class of service would have a greater rate of missed flights. Day of the week and month had intuitive effects. Relative to passengers flying on Sunday, passengers were more likely to miss flights on Monday and Friday and less likely to miss flights on other days. Mondays and Fridays are generally busier travel days and associated with more business travelers who may not personally incur the cost of missing a flight and are more prone to changing their travel plans. Taking the base month as January, passengers were more likely to miss flights in December, which is associated with heavy holiday travel and poor local weather, which may not be controlled fully with our weather dummies. This is particularly true for 2008–2009 and 2009–2010. These years experienced highly disruptive late December snow storms. Passengers were less likely to miss flights in other months. There are 230 airports included in the data set, and a number of these origins and destinations had significant fixed effects. Only the Caribbean, Canadian, and Alaskan destinations had significant regional effects relative to the continental US destinations, and these effects were negative.

Google data on flu-related internet searches were only available in weekly blocks. Different smoothing approaches were used to assign values to specific days and flights. The Google Trends search index for the key word “swine flu” generally had a positive effect on the number of missed flights and was statistically significant at the 90% confidence level, p-value = 0.072 (Table 3and 4). The result was unchanged by using mean ticket price (Table 2, rows 3–4). Only if the data were not smoothed did this pattern fail, in which case the effect of the swine flu index was not significantly different from zero (Table 2, rows 1–2).

We computed the difference between the predicted number of missed trips and the predicted number of missed trips with the coefficient associated with the Google Trends swine flu index equal to zero. This suggested that 26,393 missed trips are attributable to a behavioral response to swine flu. If U.S. Airways passengers are as likely to miss a trip because of swine flu as passengers on other airlines, then 365,717 passengers missed flights because of concern for swine flu infection or, perhaps, because they were too sick to travel – also an important behavioral consideration. This represents 0.34%, 3 to 4 in 1,000, of both the actual and predicted missed flights.

All models included destination region (Table 3 and 4) and destination city fixed effects as main effects. We repeated the estimation with two sets of interaction terms. First, we considered an interaction between destination region and the Google Trends swine flu index (Table 5). Including interaction terms had little effect on the overall model fit or the estimated coefficients presented in Table 3, and interaction terms did not qualitatively affect the estimated coefficients. However, with the exception of the interactions relating to Canada and Latin & South America all interaction terms were significantly different from zero at all common significance levels (Table 5). Moreover, the interactions terms related to Mexico, Caribbean, and Alaska were positive, and the interaction term associated with Mexico was an order of magnitude greater than the other significant interaction terms. This suggests that passengers may have been selectively avoiding these areas at times of heightened epidemic concern. This is especially true for Mexico, the epicenter of the 2009 A/H1N1 pandemic.

**Table 5:** Region interactions with the Google Trends swine flu index.

Variable	Estimate	Cluster robust Standard Error	Z-score	p-value
Google Trends swine flu index 2 week moving average	3.9×10^−4^	2.2×10^−4^	1.79	0.073
Google Trends swine flu ×				
Mexico	3.4×10^−3^	6.6×10^−4^	5.12	0
Latin & South America	6.5×10^−4^	4.9×10^−4^	1.35	0.177
Hawaii	−3.4×10^−3^	1.4×10^−4^	−24.33	0
Europe & Israel	−1.6×10^−3^	1.5×10^−4^	−10.6	0
Caribbean	7.1×10^−4^	1.5×10^−4^	4.83	0
Canada	−2.1×10^−4^	2.9×10^−4^	−0.72	0.47
Alaska	6.6×10^−4^	2.5×10^−4^	2.63	0.009

Other coefficient estimates are similar to those presented in Table 3.

We repeated the analysis to estimate interaction effects between the Google Trends swine flu index and specific destination cities. This modification had little qualitative effect on the parameter estimates, with the exception of the effect of the Google Trends swine flu index, which increased to 7.22×10^−4^. The cluster robust standard error also increased to 4.8×10^-5^ (Z-score = 1.51, p-value = 0.132). The interaction terms with Mexican destinations were all strongly significant, and of the 12 Mexican cities served by US Airways, 9 had positive effects on the number of passengers missing flights. Of the three cities with negative interaction terms, Cozumel is an island and not part of the mainland, and Guaymas and Hermosillo are the closest cities to the US border to which US Airways flys (about 400 km driving distance from the US border), while the next closest city, Mazatlan, is approximately 1,200 km from the US border.

Overall, the results suggest that some passengers skipped flights as a result of concern about a novel influenza virus called swine flu, and this behavioral adaptation was especially targeted towards Mexico.

### FluNet estimation results

The analyses were repeated using FluNet weekly A/H1N1 counts as an objective measure of the intensity of the epidemic. This is also only an index measure as underreporting is common. The estimates for all parameters from the negative binomial count model using FluNet data in the place of Google Trends data were qualitatively identical, and point estimates and standard errors were often quite close to the model using Google Trends data (Table 3 and 4) with two important exceptions. First, the estimate of the constant changed substantially, for example using the two-week moving average of the FluNet data with median price the constant term was substantially lower at 57.37 (standard error 25.63, Z-score 2.24, p-value 0.025). Second, the estimated effect of the epidemic on missed flights was nearly zero. Using the two-week moving average of the FluNet data with median price, the coefficient on FluNet case reports was −1.3×10^−5^ (Table 6). The standard error was also small, 2.0×10^−6^, yielding a Z-score 6.69 and a p-value≈0. The model suggests that the epidemic *reduced* missed flights. There is no reason that a direct response to the epidemic should cause people to miss fewer flights. We offer three possible explanations. First, people delayed travel and did not purchase tickets early in the epidemic, but then traveled later in the epidemic, which coincided with greater prevalence. Second, though unlikely, a substantial number of travelers attempted to escape the epidemic [36]. Third, and most likely, public concern is better measured by Google Trends than by the FluNet reported incidence. Therefore, the model using FluNet had to compensate for the “extra” missed flights early in the epidemic (Fig 2) by fitting a negative coefficient. Focusing on data after April 1, 2009 the correlation coefficient between the 2 week moving averages of the FluNet and Google Trends swine flu data is 0.15 (p-value<0.0001), and focusing on data from the first wave, when the Google Trends index was greatest (4/1 to 8/1 of 2009), the correlation coefficient between the two epidemic indices was negative (*R* = −0.21, p-value<0.0001). This pattern holds regardless of the smoothing process.

**Table 6:** Model specification robustness results for FluNet models.

Length of moving average	Price index	Regional interaction with flu index	City interactions with flu index	Log pseudo- likelihood	Estimate of flu index coefficient	p-value for coefficient
1	median	no	no	−4022174	−7.46×10^−6^	0.000
1	mean	no	no	−4022365	−7.15×10^−6^	0.000
2	median	no	no	−4022024	−1.3×10^−5^	0.000
2	mean	no	no	−4022220	−1.3×10^−5^	0.000
3	median	no	no	−4021695	−2.2×10^−5^	0.000
3	mean	no	no	−4021897	−2.2×10^−5^	0.000
1	median	yes	no	−4022142	−7.20×10^−6^	0.000
1	mean	yes	no	−4022334	−6.91×10^−6^	0.000
2	median	yes	no	−4021993	1.91×10^−6^	0.000
2	mean	yes	no	−4022190	−1.3×10^−5^	0.000
3	median	yes	no	−4021666	−2.2×10^−5^	0.000
3	mean	yes	no	−4021867	−2.1×10^−5^	0.000
1	median	no	yes	−4021583	1.02×10^−5^	0.026
1	mean	no	yes	−4021781	1.04×10^−5^	0.021
2	median	no	yes	−4021432	7.01×10^−6^	0.084
2	mean	no	yes	−4021635	7.25×10^−6^	0.069
3	median	no	yes	−4021103	−4.16×10^−7^	0.946
3	mean	no	yes	−4021311	−1.85×10^−7^	0.976

When days are assigned there epidemic index value from weekly data, the length of moving average is listed as 1. Price index lists whether median or mean prices were used, the interactions columns state whether interactions were included.

Generalizing the model beyond main effects to include a set of FluNet cases-by-destination region and case-by-city interaction terms had no qualitative effect on parameter estimates, with one important exception: for some specifications the FluNet case coefficient was significant and had a positive sign, but in these cases the coefficient remained relatively small in magnitude (Table 6). Taken together, these models suggest that passengers did not skip flights in response to reported A/H1N1 cases. Furthermore, evidence that people responded to Google Trends, but not to an index of actual cases, suggests that those missing flights in response to the Google Trends swine flu index engaged in avoidance behavior and were not missing their flights because they were sick.

### Willingness to pay to avoid infection

A lower bound on the per flight cost of skipped trips is defined by the median price paid for that flight multiplied by the number of passengers expected to miss that flight out of concern for swine flu. For U.S. Airways passengers this implied a willingness to pay ranging between $2.67 million when city level interaction terms are included and $3.61 million for the base model. If the base model prices are representative, then the willingness to pay to eliminate infection risk during travel was at least $50.1 million nationally. These calculations represent a lower bound on what passengers would have been willing to pay to eliminate the chance of contracting A/H1N1 swine flu while flying. These calculations do not represent total losses to the airline industry or losses to society at large from people choosing not to purchase a ticket as a result of H1N1 swine flu. It is likely that people simply postponed travel decisions, like other consumption decisions, until the epidemic waned [37].

Our results suggest that travelers responded to concern about the epidemic, but not in a way that matched the actual incidence of infection. It is unlikely that epidemics such as the 2009 A/H1N1 outbreak can be completely avoided, but it is possible to better communicate risks. Fig 2 suggests that early in the epidemic public concern was disproportionate to the number of cases. Indeed, the public may have been panicked by vice president Biden's advice to skip trips (http://abcnews.go.com/Politics/story?id=7470281&page=1#.UAn0EaNdC1c). To bound the cost of this “adaptive” panic we rescaled the Google Trends swine flu index by 235 (as in Fig 2) and adjust the associated parameter in the models that use the Google Trends data. These adjustments are unit conversions. The rescaling is necessary because the units of the Google Trends are arbitrary. The number 235 was chosen to align the Google Trends data and the FluNet data during the second wave of the epidemic. We then forecast the number of missed trips and a lower bound on the cost of these missed trips relative to a counterfactual scenario where public concern tracked the actual number of cases. Our base model suggested that US Airways passengers missed 13,507 excess trips as a result of concern in excess of that merited by case reports alone, resulting in at least $1.7 M in forgone travel benefits. Scaling to the United States air traveling population implies 187,078 missed flights in excess of what would have been missed had public concern tracked actual cases and $24.1 M in forgone travel benefits – nearly half the lower bound value of completely eliminating the epidemic. The model with a destination city interaction suggested that an additional 160,387 trips would have been taken had public concern matched cases resulting in $18.5 M in forgone travel benefits.

## Discussion

Behavioral changes can potentially have a large effect on epidemic dynamics [2], [3], [28], [38]. There is scarce evidence on the economic tradeoffs individuals make to preserve their health during an epidemic [39]. Most research in this area, particularly with respect to behavioral change and air travel, has relied on survey data using hypothetical scenarios ([e.g., [40], [41], [42]) and not revealed behavior. Choosing to miss a flight is a costly decision. The median cost of a round trip flight in our data set is approximately $300. Nevertheless, we find some individuals change their behavior in response to a population level measure of concern about a novel pathogen. This is not all together surprising; vice-president Biden recommended that people avoid airplanes and subways during an interview on NBC's Today show.

Mao [28] suggests that self-imposed behavioral changes during an epidemic may be a low cost way to control disease. However, Mao assumes that the perceived risk maps to actual risks. Early in the A/H1N1 epidemic the expected costs from contracting that pathogen may have been high and declined as it was learned that the pathogen was less severe. Due to underreporting and potential misreporting, reported cases are likely an inexact measure of risk, but may map to objective measures of risk. The data suggest that the public did not respond to the cases, but perhaps to media attention to the epidemic. If cases are a good measure of objective risk, then nearly half the costs to air passengers could have been avoided through clearer communication about health risks.

It is not clear that the early response was irrational. The observation that the two flu indices primarily diverge early in the epidemic suggests that most behavioral changes happened in the early days of the epidemic. Fenichel and Wang [22] suggest that epidemiological forecasts based on reproductive number theory (e.g., R0) may lead to excessive risk reducing behavior if the estimators do not account for how behavioral responses affect the epidemic. Reproductive number theory is the standard in modern epidemiology, and there were many attempts to forecast the A/H1N1 epidemic using reproductive number theory early in the epidemic ([e.g., [43], [44], [45]). Developing the next generation of epidemiological forecasting tools requires integrating estimation of human responses to disease risk with estimation of the basic epidemiological parameters [2]. The current paper advances that goal by providing a quantitative analysis of human behavioral responses to a rapidly disseminating disease.

An important policy question is where to invest scarce resources to reduce the economic damages of an epidemic. It is important that public health measures are less costly than the epidemic itself [37], [46]. We do not know the cost of improving risk communication, but our calculations suggest that better communication of actual risk can provide substantial cost savings. For example, the U.S. Environmental Protection Agency has developed AirNow.gov, an online site and mobile app that provides real time spatially explicit air quality data to help people make decisions about outdoor activities that may interact with air quality to affect health. Others are developing similar applications for infectious disease.

Finally, air travel has been targeted as a venue for non-pharmaceutical interventions [9]. We estimate the willingness to pay by the air traveling public to eliminate the risk of contracting swine flu while traveling to be on the order of at least $50 M over the two year period bracketing the swine flu outbreak. In comparison, Epstein et al. [10] estimate the cost of a complete US air travel shut down in terms of lost consumer surplus to prevent flu to be $93-$100B/yr. Our estimate does not consider the willingness to pay of non-travelers to reduce the probability that air travelers will spread the disease more quickly. However, [11]–[13] argue that air travel restrictions at best are likely to have modest effects on the spread of infectious disease. Airport screening programs represent a non-pharmaceutical intervention. Bitar et al. [18] review the literature on the use of infrared detection systems in airports to detect travelers with fever, and find these systems to be ineffective. Dell'Omodarme and Prati [47] suggest that from a statistical standpoint such surveillance is unlikely to be effective. If such scanners could be improved to fully prevent passengers from catching flu, then given our lower bound estimates and the current price of $2,500 per scanner [48], one could purchase just over 20,000 scanners to serve the United States' approximately 430 commercial airports, approximately 50 per airport not including labor costs or the costs of passenger delays. This number would be approximately cut in half if communication strategies were improved first. This suggests that technological innovation in flu scanning could, in principle, be cost effective, but clear risk communication and accounting for the public response to this information may be more cost effective, especially in the short to medium run.

Our findings suggest that people do respond to epidemiological risks with behavioral change. The effect of these behavioral responses on epidemic spread is an area of ongoing research. Furthermore, the nature of the feedback from disease spread to human behavior appears to be tightly connected to information about the epidemic, and that information may only be loosely connected to facts on the ground. This “noise” complicates developing forecasting models that account for behavioral-epidemiological feedbacks, which remains an important area for continued research. Nevertheless, the results suggest a clear need to enhance risk communication strategies related to infectious diseases. Infectious diseases are scary, but clear communication appears to have substantial potential to lessen the hardships caused by an epidemic.
